# Surgical approach for complete cochlear coverage in EAS-patients after residual hearing loss

**DOI:** 10.1371/journal.pone.0223121

**Published:** 2019-09-26

**Authors:** Nora M. Weiss, Anandhan Dhanasingh, Sebastian P. Schraven, Marko Schulze, Soenke Langner, Robert Mlynski

**Affiliations:** 1 Dept. of Otorhinolaryngology, Head and Neck Surgery,”Otto Koerner”Rostock University Medical Center, Rostock, Germany; 2 MED-EL, Innsbruck, Austria; 3 Rostock University Medical Center, Institute of Anatomy Gertrudenstraße, Rostock, Germany; 4 Institute of Diagnostic and Interventional Radiology, Pediatric and Neuroradiology, Rostock University Medical Center, Rostock, Germany; University of Porto Faculty of Medicine, PORTUGAL

## Abstract

**Introduction:**

In cases with residual-hearing (RH) loss after cochlear implantation, a safe method is needed to provide full spectral resolution and as much auditory information as possible without implant replacement. Aim of this study was to prove the feasibility of accessing a partially inserted cochlear-implant-electrode for complete insertion to its maximum length through the external ear canal using a transcanal approach.

**Methods:**

Two CI electrodes were customized with 18 stimulating channels. The electrode design enables the use of 12 active channels available for electrical stimulation inside the cochlea both after partial and full insertion. 10 CI electrodes were implanted in 10 fresh human cadaveric temporal bones. After initial partial insertion by posterior tympanotomy, the electrode was inserted to its maximum length via a transcanal approach. Radiographs and CT scans were performed to confirm the electrode position. The electrodes were investigated via x-ray after removal.

**Results:**

X-ray and CT-scans confirmed the electrode prototypes covering an angular insertion depth between 236° to 307° after initial insertion. Accessing the electrode in the middle ear space was feasible and insertion to its full length was successful. Post-insertion CT confirmed insertion of the 28mm and 31.5mm electrode arrays covering an angular insertion depth between 360° and 540° respectively. No tip foldovers were detected.

**Conclusion:**

This study confirms the feasibility of extending the electrode insertion to its maximum insertion length using a transcanal approach in temporal bone specimens. This constitutes a second stage procedure on demand in EAS-surgery. This may be beneficial for EAS-patients providing electrical stimulation beyond the basal turn of the cochlea once the functional residual hearing is lost, without replacing the entire CI.

## Introduction

Hearing and Structure Preservation (HSP) has become a topic of general interest in the Cochlear Implantation (CI) field even in cases where the low frequency residual hearing is not functionally benefitting the patients [1]. CI electrodes have undergone significant design changes over the years mainly focusing on structure preservation. A higher degree of intracochlear trauma was observed using precurved modiolar-hugging electrodes compared to straight lateral wall electrodes [2].

Patients successfully using Electro-Acoustic-Stimulation (EAS) have shown to achieve better hearing outcomes than patients being electrically stimulated (ES) only with shorter electrode arrays [3]. Though long and flexible electrodes have shown to preserve the low frequency hearing [4], yet short electrodes are widely considered being advantageous in preserving the residual hearing (RH) especially in EAS cases. HSP surgery in EAS cases has shown to provide mixed results with some patients losing the residual hearing (RH) immediately postoperatively [5] while some patients preserve it years after surgery [4]. The exact mechanism that preserves the RH is still to be understood. There are reports available that a very short electrode could not provide the full benefit of the CI to the patient, especially when the residual hearing is lost completely requiring revision surgery with a standard length electrode [6,7] for providing electric stimulation beyond the basal turn of the cochlea and complete cochlear coverage.

Electrode selection for EAS surgery is challenging as the surgeon has to plan well how to treat the patient if the functional RH is lost postoperatively. In case of RH loss postoperatively, revision surgery implanting a new implant with a standard length electrode for complete cochlear coverage causes additional trauma to the patient and is cost-intensive. Inserting a regular electrode array partially only with the option of full insertion is one approach to address the problem of RH loss [8]. However, partial insertion reduces the spectral resolution to the number of electrodes inserted and therefore provides less auditory information than principally possible. With this background, it was the aim of this study to investigate whether it is achievable to access a modified CI electrode array with increased spectral capacity through the external auditory canal into the middle ear space. This surgical approach would provide a comprehensive way of improving the auditory information of EAS patients, when the functional RH is lost post-operatively. The method may avoid invasive and expensive revision surgery that requires a new implant.

## Material and methods

### Ethical consideration

Fresh temporal bones with intact middle ear structures were taken to perform this feasibility study. They originated from the University donor program at the Anatomical Insitute of Rostock University. All patients gave written informed consent during lifetime for scientific investigation (ethics board: Ethics committee of the medical faculty of the University of Rostock; St.-Georg-Str. 108; 18055 Rostock). According to German laws, a person wishing to donate his/her body for medical research after death should register voluntarily with the program during lifetime. The donor’s privacy was protected. None of the participants in this study had any identifying information such as the name, gender or age of the donor, nor had they access to the database of the willed body donation program.

### Electrode design

**Fig 1:**
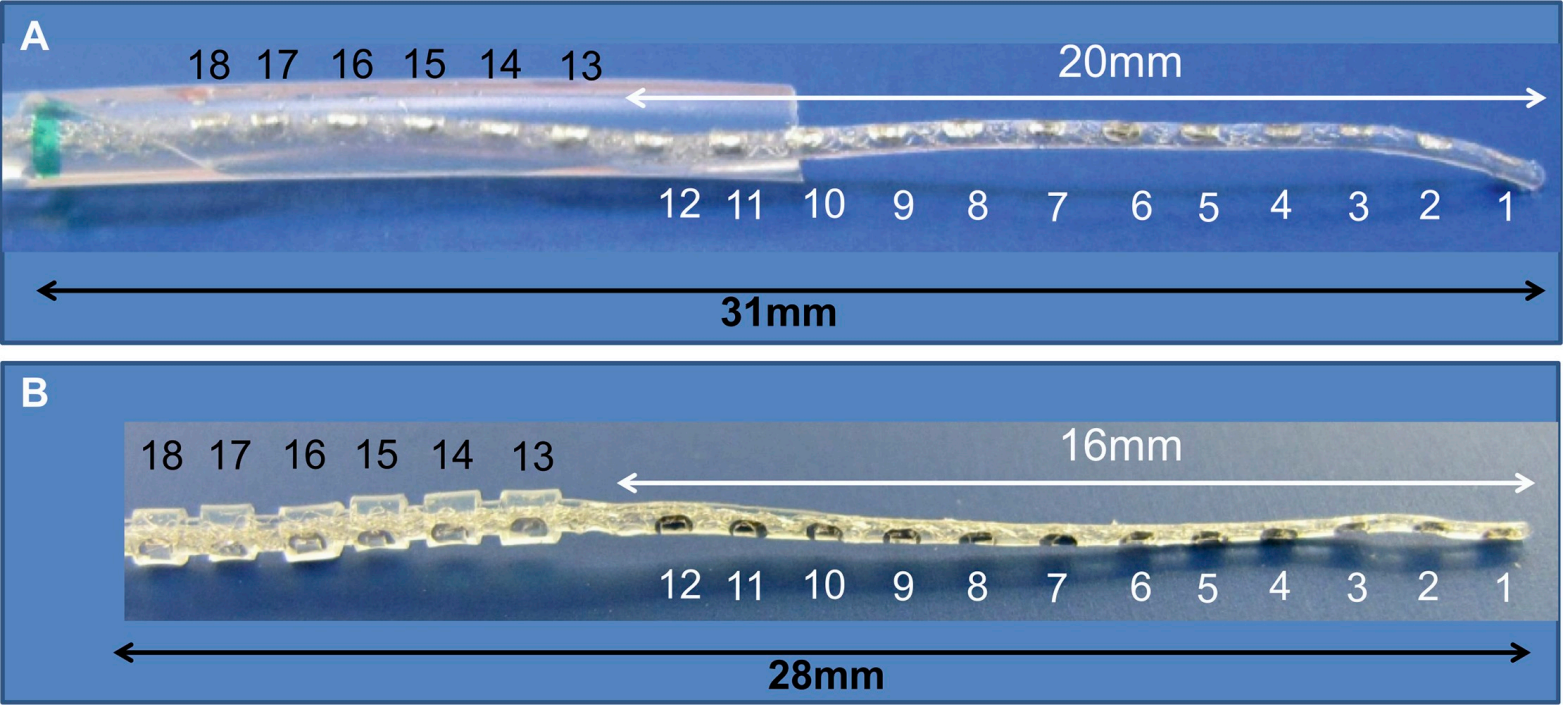
Electrode-prototypes: **A:** Electrode-1 has an overall array length of 31mm with the first 12 stimulating channels covering a length of 20mm. This prototype has the insulation in the form of a simple cylindrical tube that can easily be moved up and down the array. **B:** Electrode-2 has an overall array length of 28mm with the first 12 stimulating channels covering a length of 20mm. This prototype has the insulation in the form of individual silicone rings tightly covering the external 6 stimulating channels.

Two different electrode array lengths, one with 31.5mm and the other with 28mm were manufactured on custom request by MED-EL (Innsbruck, Austria) (Fig 1). The electrode-1 had the first 12 stimulating channels in the apical 20 mm length for the partial insertion and additional 6 stimulating channels in the basal 11mm temporarily covered by a tubular sheath for the full insertion. The electrode-2 had the first 12 stimulation channels in the apical 16 mm length for the partial insertion and the additional 6 stimulating channels in the basal 12 mm covered each by a tight silicone ring for the full insertion. Implants/electrodes that are commercially available by this manufacturer are engineered overall with 12 independent stimulating channels. A future insertion of the additional 6 stimulating channels should be balanced by deactivating 6 alternating channels from the apical portion that were initially inserted so that the number of stimulating channels is still 12 and not 18. This can be achieved by the special electrode design that commands an external loop carrying the wires of the 6 alternating channels from the apical section. Cutting this loop deactivates those channels in case of RH loss and consecutive completed full insertion. Fig 1 shows the two different prototypes of electrodes.

### Surgical description

The experiment was performed by 3 surgeons with different grades of experience in cochlea implantation (professional (RAM) n>600, intermediate (SPS) n = 100, beginner (NMW) n = 0). The experiment was carried out in two steps, first investigating the more appropriate electrode design. Electrode-2 was considered to be superior to electrode-1 due to its singular insulated electrodes with individual silicone rings that enabled a better overview during the procedure. The second part of the experiment was carried out with electrode-2 only. Eight additional electrodes were produced in order to have 3 electrodes of this prototype to be implanted by each surgeon. Prototype 1–4 (1 x electrode-1, 3 x electrode-2) were inserted by the professional, 5–7 by the intermediate and 8–10 by the beginner.

***Step 1*:** Standard mastoidectomy and posterior tympanotomy were performed along with the resection of the bony overhang of the oval window niche in order to clearly identify the round window membrane. The two different electrodes were inserted via the round window (RW) membrane until the first 12 electrodes were placed inside the cochlea followed by an X-ray (Philips Bucky Diagnost, Philips Healthcare, Hamburg, Germany) in Stenver’s view to determine the initial electrode insertion length. The procedure was repeated with electrode-2 in 8 additional temporal bones and CT-scan was performed to document partial insertion. All CT examinations were performed on a 64-row CT scanner (Aquillion 64, Canon Medical, Tustin, CA, USA; representative CT imaging protocol: 120 kV, 150 mA). All CT dataset were reconstructed in axial and coronal planes in bone window/level settings with a standard field-of-view (FoV; 22cm) with a slice thickness of 1mm and a slice gap of 0.5mm.

**Fig 2:**
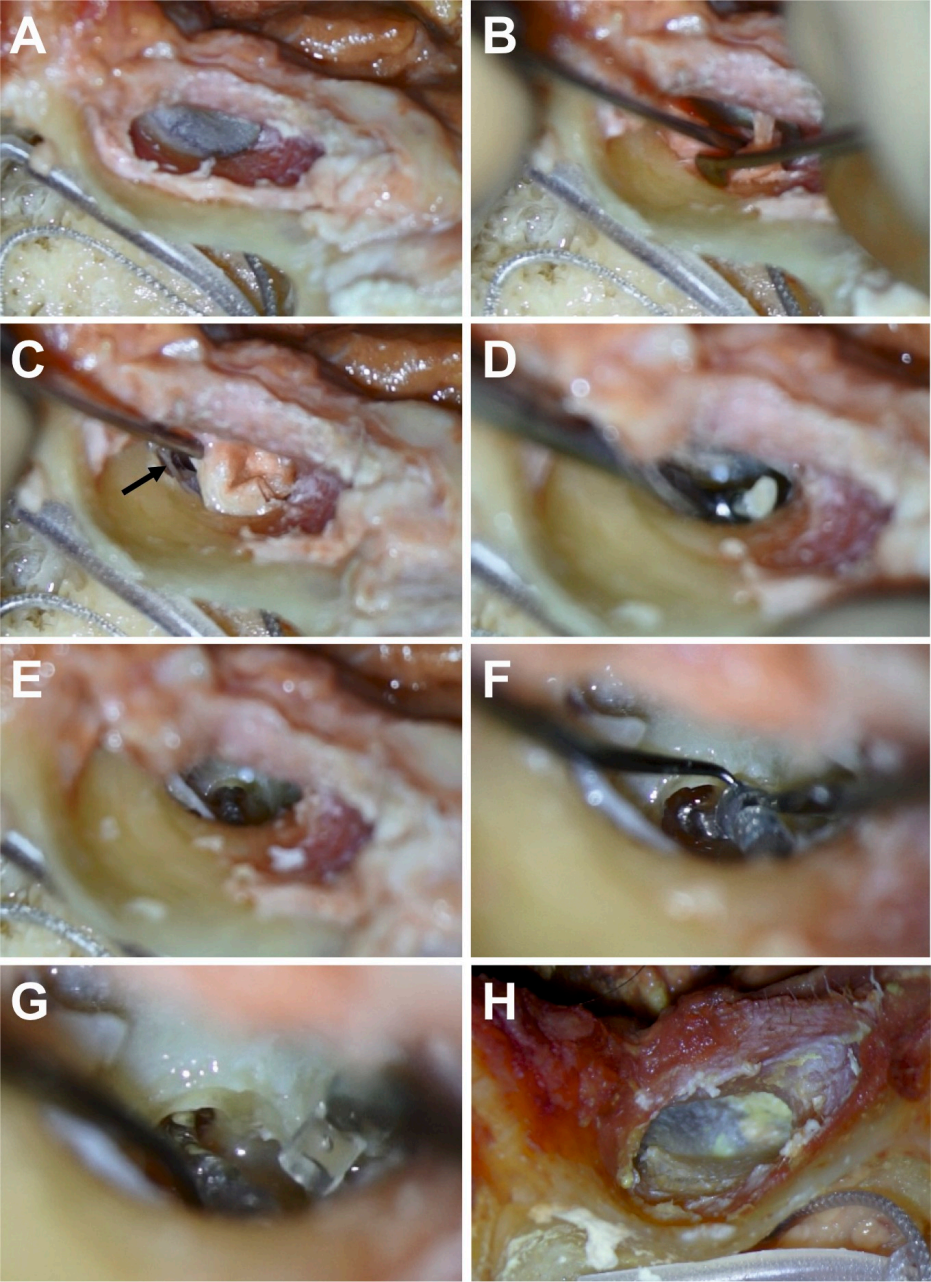
Surgical steps involved in opening the eardrum to access the electrode in the middle ear space. **A:** Assessing the middle ear using a transcanal approach; **B/C**: Opening the middle ear and visualizing the Chorda tympani nerve (➞); **D**: Partial removal of the lateral attic wall using a House curette; **E**: Identification of the electrode using a transcanal approach; **F**: Removing the insulation ring from the external channels; **G**: Maximal insertion of the electrode inside the cochlea; **H**: Repositioned tympanic membrane.

***Step 2*:** Afterwards, the middle ear was accessed using a transcanal approach as it is used in standard tympanoscopy (Fig 2A). The meatal skin was incised. The posterior 180° segments of the tympanic membrane were lifted to enter the middle-ear by raising the fibrous annulus and incision of the mucosa that connects the tympanic membrane to the bone. The chorda tympani nerve which is in the field of view was identified and preserved. The procedure was performed using a surgical microscope (iView 31, Atmos, ORT) (Fig 2B and 2C). For appropriate exposition of the promontory and the round window niche, the bone of the lateral attic wall was removed using a House curette (Storz, Tuttlingen, Germany) (Fig 2D). Bone removal was sufficient if the pyramidal eminence and the inferior margin of the round window could be identified in a binocular view. After removal of the insulation of the external 6 electrodes (Fig 2F), the electrode was inserted to its maximum inside the cochlea using standard otologic microsurgical equipment (Fig 2G). The surgery was followed by a clinical CT-scan of the temporal bone using the above described protocol to determine the final electrode position in all specimens (Aquillion 64, Canon Medical, Tustin, CA, USA). All CT datasets were reconstructed in axial and coronal planes in bone window/level settings with a slice thickness of 1mm and a slice gap of 0.5mm (120 kV, 150 mA).

### Statistical analysis

All statistical tests were selected before data collection. Data was transferred to standard data spread sheets and statistical analyses performed using Microsoft Excel (Version 15.29, Microsoft, Redmond, Washington, USA) and Prism (version 7, GraphPad Software, La Jolla, CA, USA). The significance level was set to p < 0.05.

## Results

The main aspect that was focused on in this study was the evaluation of accessing the middle ear space through the external ear canal using standard surgical steps. First the feasibility of removing the insulation material covering the additional six external channels was evaluated. The second issue of interest was the feasibility of pushing the electrode to its maximum insertion depth.

**Fig 3:**
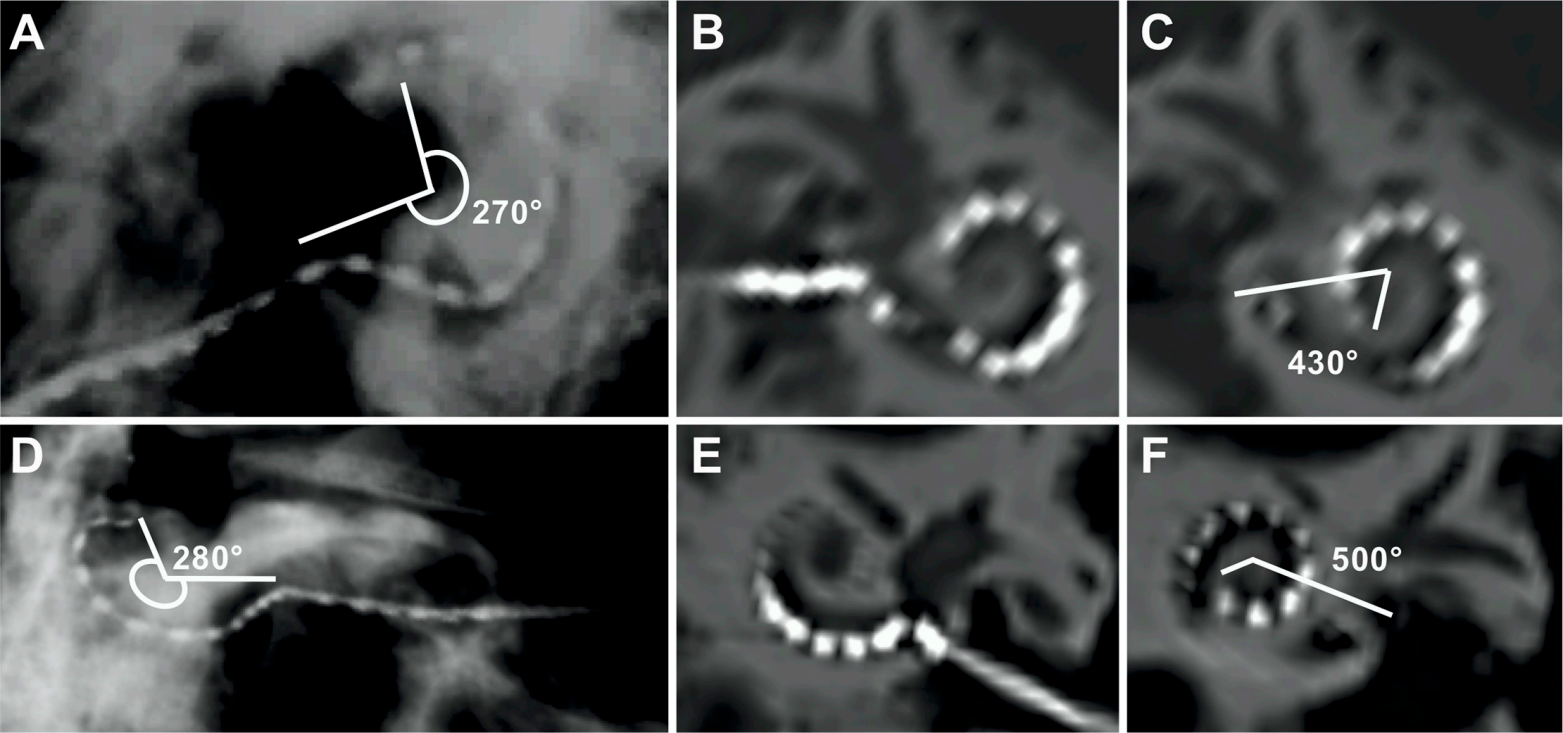
Insertion depth and angle of the first insertion and later insertion with both electrodes: **A**: X-Ray in Stenver’s view after partial insertion of Electrode-1; **B**: CT-scan after full insertion, showing an insertion angle of 430° for Electrode-1 (**C**); **D**: X-Ray in Stenver’s view after partial insertion of Electrode-2; **E**: CT-scan after full insertion, showing an insertion angle of 500° for Electrode-2 (**F**).

The partial insertion through the posterior tympanotomy/facial recess of the first 20 mm and 16 mm array length having 12 stimulating channels from both the electrode prototypes respectively covered an angular insertion depth of 270° and 280° as shown by the x-ray pictures given in Fig 3A and 3D. Through the external ear canal after opening the tympanic membrane, the electrode was assessed for further advancement inside the cochlea.

The design of electrode-1 provided a cylindrical tube that was covering the additional channels outside the cochlea. It was easily retracted when advancing the electrode further inside the cochlea. Electrode-2, was designed with individual silicone rings that were tightly covering the six external stimulating channels. Each was individually removed, using a sickle-knife, followed by a one-by-one insertion inside the cochlea. Electrode-2 was much slimmer than electrode-1, giving some insertion resistance for electrode-2 when approaching full insertion. After full insertion of the electrode, the tympanic membrane and external ear canal skin were repositioned. The post-surgical CT scans in the reconstructed coronal view confirmed deeper insertion of both the electrode prototypes covering an angular insertion depth of 430° and 500° respectively with no tip foldover (Fig 3C and 3F). Electrode-1 was almost fully inserted leaving the last 2 channels outside the RW entrance while electrode-2 had the last channel positioned inside the RW entrance. CT-scan showed a sharp bending of electrode-2 inside the RW entrance (Fig 3B and 3E).

Electrode-2 was considered advantageous due to its slim design and the single insulation rings. In cases where full insertion cannot be achieved, it provides coverage of the electrodes, remaining outside the cochlea without the risk for facial excitation. Even if further insertion of the electrode is not possible to its full extent, electrodes staying outside the cochlea remain covered. Activating the inserted electrodes by cutting the loop is still possible even when electrodes are not fully inserted. For this reason, the experiment was continued with prototype-2-electrodes only.

**Fig 4:**
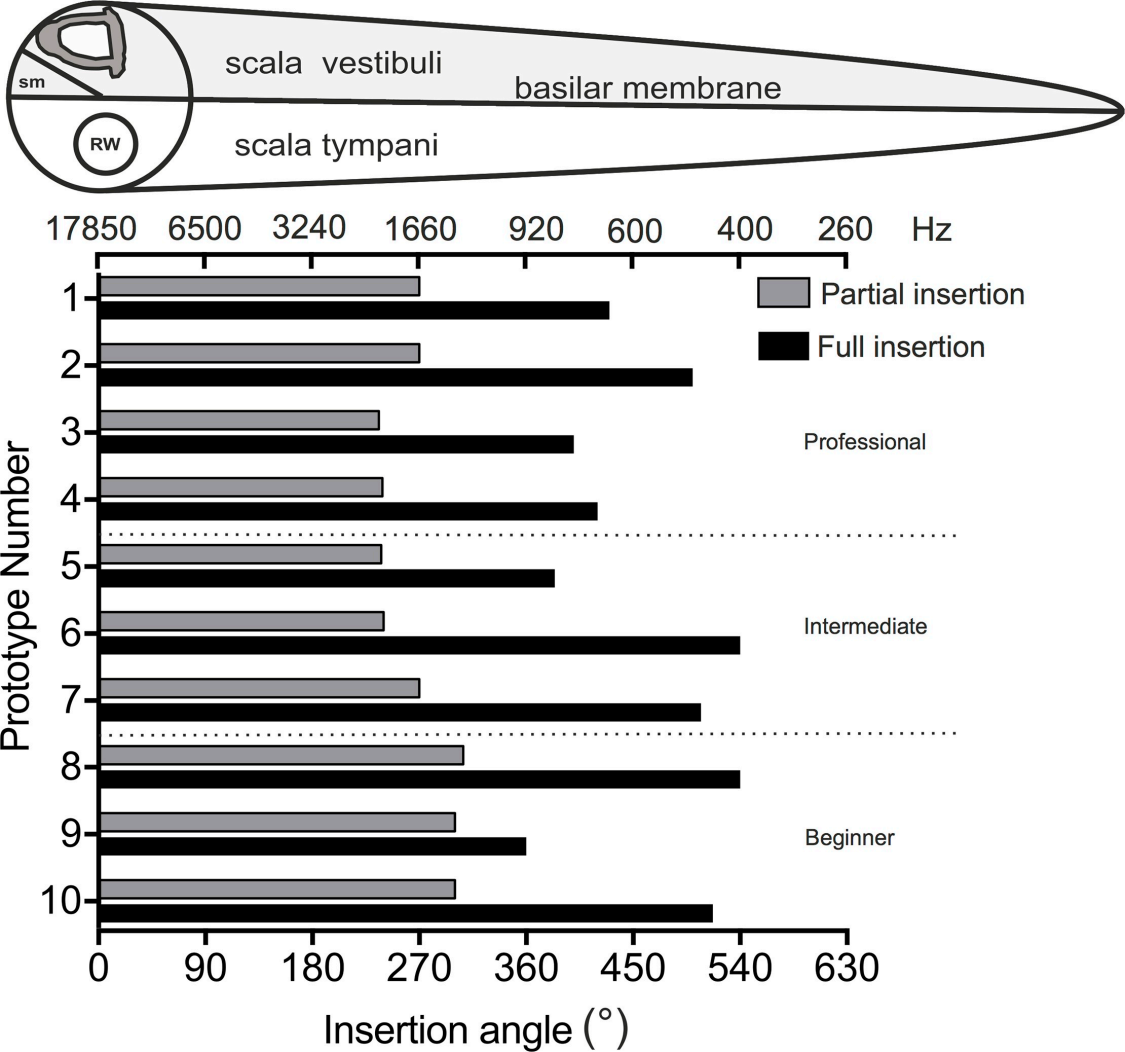
Insertion depth and angle of all 10 Prototypes at partial and full insertion. RW = round window, sm = scala media, frequency bar indicating the approximate frequency according to the insertion depth [17].

In 5 cases (50%) a full insertion was not successful. Between 1–5 electrode-contacts were left outside the cochlea due to resistances during the insertion, a disadvantageous insertion angle or due to damage to the electrode (Prototype 1: 2 electrode contacts; Prototype 3: 3 electrode contacts; Prototype 4: 1 electrode contact; Prototype 5: 3 electrode contacts; Prototype 9: 5 electrode contacts). Fig 4 shows the insertion depth and angle of all 10 Prototypes at partial and full insertion. The chorda tympani was damaged in 3 cases (30%) (Prototype 3, 7, 10).

**Fig 5:**
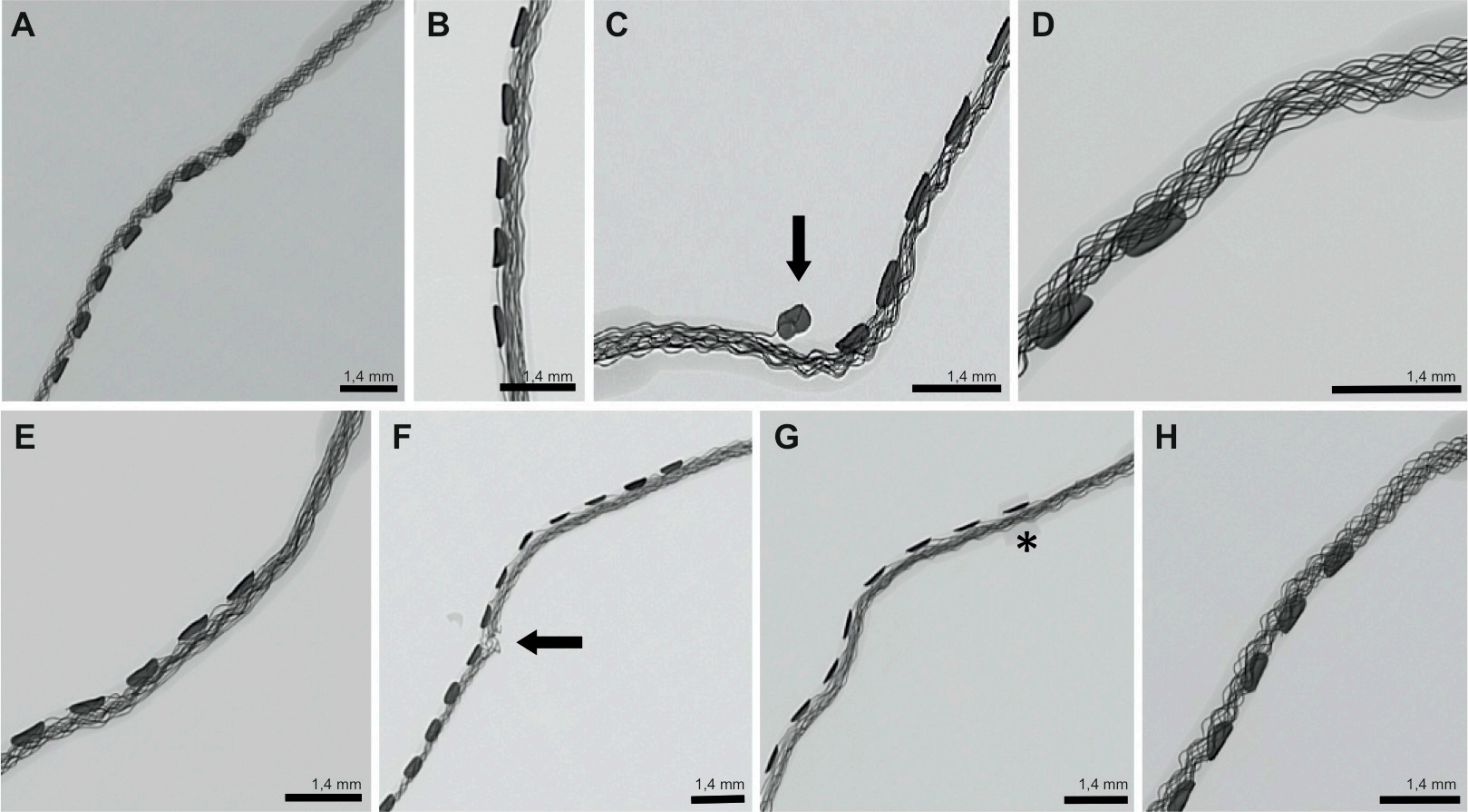
Micro-X-ray of the electrode-Prototypes 3–10 after removal; the arrows marking the wire-damage of electrode 5 and 8. **5A**: Prototype 3: no relevant damage, slight kinking at the very base; **5B**: Prototype 4: no damage, no kinking; **5C**: Prototype 5: showing a very high kinking and the detached contact at the base at Prototype 5; **5D**: Prototype 6: slight kinking at the base; **5E**: Prototype 7: slight kinking at the base; **5 F**: Prototype 8: the electrode array of Prototype 8 was cut; **5 G**: Prototype 9: the last insulation ring of Prototype 9 is still in place—Asterisk); **5 H**: Prototype 10: no damage, no kinking.

No differences between the surgeon’s degree of experience could be observed concerning chorda-tympani-preservation, insertion-time and insertion-depth. 2 Prototypes (Prototype 5 and 8) were damaged during the removal of the insulation rings. In both cases it was the first attempt of the surgeon and happened to the intermediate and the beginner only. No wire-damage was observed at the electrodes inserted by the professional surgeon. Fig 5 shows the X-rays of the implants after removal.

The mean time for full-insertion using the transcanal approach was 13 minutes (16 minutes for the professional, 8 minutes for the intermediate and 15 minutes for the beginner). No significant correlation between the duration of insertion and the insertion depth or the damage of the chorda tympani nerve could be found.

## Discussion

The standard of care for sensorineural hearing loss is improving with the advancement of CI development, especially concerning the electrodes. Also, the surgical procedures are getting as minimally invasive as possible both, for the patient’s comfort and the aesthetic look [9]. Nevertheless, hearing preservation cannot be achieved in every case [10] though the reasons for RH-loss are still to be understood. Additionally, in every case of cochlear implantation remains a risk of progressive hearing loss over time [11]. In case of perioperative RH loss, when only electric stimulation can be achieved, the electrode length seems to play a role concerning the speech-performance [3]. For the patient’s as well as for the surgeon’s contentment, it is desirable not to have a cost- and pain-intensive revision surgery due to a disadvantageously chosen electrode at initial surgery. Apart from the length, it has been found, that also the position to the modiolus and the approach to the cochlea play a role in hearing preservation in favor for straight electrodes and RW-insertion [12,13]. Choosing the right electrode variant in EAS-surgery is a major task and the question whether to choose a shorter electrode for preserving the residual hearing or a longer electrode under the aim of better speech performance in case of progressive hearing loss is discussed regularly [14]. In case of immediate post-operative or progressive hearing loss after cochlear implantation, additional cochlear coverage can be beneficial for a better hearing gain. Fitzgerald et al. 2008 and Carlson et al. 2012 showed, that especially in cases with short hybrid-electrodes, reimplantation with a standard electrode resulted in rapid improvement of speech perception after a time of up to 6 months [6,7].

Providing a method for achieving the maximum cochlear coverage with electrical stimulation that can be performed in a short surgical procedure with the same electrode not only reduces the patients’ surgical risk but also the costs to the healthcare system caused by, hospitalization and electrode-/implant substitution. The surgery can be potentially performed under local anesthesia in selected patients. Without any change in the current electrode design, one option for avoiding future electrode exchange would be to use a regular standard electrode with only partial insertion of some number of stimulating channels inside the cochlea and deactivating the remaining number of channels that are extra-cochlear [8,15]. This may lead to a spectral reduction due to less channels than possible and not providing the full benefit of the CI to the patient under the assumption that a greater number of stimulating channels provides better hearing.

Approaching this task, the electrode prototypes and the surgical technique presented in this study enable the stimulation of the full number of the 12 channel implant inside the cochlea at any given situation. Consequently, the patient fully benefits the device. Though it is commonly discussed that complete insertion of long electrodes inside the cochlea is challenging in a cadaveric temporal bone setting, it was possible to show that full insertion or close to full insertion is possible.

This is the first study evaluating the feasibility of further advancing an electrode array from the middle ear region into the cochlea, accessing the electrode through the external ear canal by opening the tympanic membrane. Arguably, fibrous encapsulation may lead to a fixation of the electrode inside the mastoid in a clinical setting that impedes the protrusion. Even though this temporal bone experiment is not able to simulate any fibrous tissue around the excess electrode, we consider this issue negligible, since the aeriation of the mastoid is supported by the antrotomy. Antrotomy is the first step of transmastoid cochlea implantation exposing the landmarks of the middle cranial fossa and the short incus process followed by the subtotal mastoidectomy and posterior tympanotomy. This procedure is also undertaken in chronic otitis media surgery to improve the aeriation of the mastoid in various otological schools. For this reason, we consider the antrotomy beneficial in CI-patients to guarantee the aeriation of the mastoid. Additionally, the important functional part of the electrode containing the additional contacts predominantly is located in the tympanic cavity which is accessible for the surgeon. Manrique-Huarte et al. proved, that progressive insertion of the electrode to full insertion is feasible after 3 months, even in cases with fibrous or ossification tissue in an animal experiment with Macaca fascicularis primates [16]. Fibrous formation around the electrode may prevent further advancement of the electrode in patients with longer periods of residual hearing loss. It might be advisable to revise the electrode position in the cochlea as early as possible after residual hearing loss.

We observed, that there was a training effect, since in each case, where the electrode was damaged it was the first insertion for the corresponding surgeon. After a training-period in the temporal bone laboratory, we consider the method save, even for unexperienced surgeons. The same applies to the rather high rate of damage to the chorda tympani. In case of using this method in a clinical setting, training this approach in temporal bones is recommend.

The insulation rings were tightly attached to the electrodes, impeding the removal and like that leading to electrode damage in 2 cases. A close attachment is necessary to ensure a safe insulation and stable position. But since the implementation of the instruments for a safe removal can easily be trained in the temporal bone laboratory, we don’t consider this fact as disadvantageous.

Since the experiment was performed in cadaveric bones, we observed the problem of not achieving full insertion in 5 cases. In those cases, were no full insertion is achieved, the new electrode design with single insulation rings covers the opportunity to leave part of the electrode outside the cochlea, still profiting from the additionally inserted electrodes and not risking a co-excitation of the middle ear structures including the facial nerve.

The presented method could be performed under local anesthesia very much similar to stapes surgery. Depending on the surgical school, patients’ risk factors and preferences, stapes surgery is performed under local anesthesia allowing manipulation to the oval window and is well tolerated. This leads to a reduction of the patient’s morbidity and inpatient time. This issue may be advantageous especially for elderly patients suffering from risk-factors for general anesthesia. We expect this feasibility study to encourage more surgeons and manufacturers to consider this method for treating EAS cases. Hesitation in the indication of EAS surgery will be less if appropriate treatment options in case of residual hearing loss are available. Thus, more patients receive rehabilitation and health care costs can be reduced. Applying this procedure in clinical practice along with recommending the CI companies to advance the electrode designs may evolve future EAS-systems.

## Conclusions

This experiment proved the feasibility of further advancing an electrode array from the middle ear into the cochlea, accessing specially designed EAS electrodes through the external ear canal. This procedure can highly reduce the cost, time and morbidity associated with revision surgery under general anesthesia in case of postsurgical or progressive hearing loss. We assume that the presented study helps advancing the standard of care for hearing loss in patients receiving cochlear implantation.

## Supporting information

S1 Table Insertion depth of numbered electrodes after first and second insertion. (XLSX)

## Abbreviations

CI: Cochlear Implantation
CT: Computed Tomograph
EAS: Electro-Acoustic-Stimulation
ES: Electric Stimulation
HSP: Hearing and Structure Preservation
RH: Residual Hearing
RW: Round Window
